# Restoration of Muscle Glycogen and Functional Capacity: Role of Post-Exercise Carbohydrate and Protein Co-Ingestion

**DOI:** 10.3390/nu10020253

**Published:** 2018-02-23

**Authors:** Abdullah F. Alghannam, Javier T. Gonzalez, James A. Betts

**Affiliations:** 1 College of Medical Rehabilitation, Qassim University, Buraidah 51451, Saudi Arabia; 2 Human Physiology Research Group, Department for Health, University of Bath, Bath BA2 7AY, UK; j.t.gonzalez@bath.ac.uk (J.T.G.); j.betts@bath.ac.uk (J.A.B.)

**Keywords:** post-exercise, sports nutrition, recovery, glycogen, subsequent exercise

## Abstract

The importance of post-exercise recovery nutrition has been well described in recent years, leading to its incorporation as an integral part of training regimes in both athletes and active individuals. Muscle glycogen depletion during an initial prolonged exercise bout is a main factor in the onset of fatigue and so the replenishment of glycogen stores may be important for recovery of functional capacity. Nevertheless, nutritional considerations for optimal short-term (3–6 h) recovery remain incompletely elucidated, particularly surrounding the precise amount of specific types of nutrients required. Current nutritional guidelines to maximise muscle glycogen availability within limited recovery are provided under the assumption that similar fatigue mechanisms (i.e., muscle glycogen depletion) are involved during a repeated exercise bout. Indeed, recent data support the notion that muscle glycogen availability is a determinant of subsequent endurance capacity following limited recovery. Thus, carbohydrate ingestion can be utilised to influence the restoration of endurance capacity following exhaustive exercise. One strategy with the potential to accelerate muscle glycogen resynthesis and/or functional capacity beyond merely ingesting adequate carbohydrate is the co-ingestion of added protein. While numerous studies have been instigated, a consensus that is related to the influence of carbohydrate-protein ingestion in maximising muscle glycogen during short-term recovery and repeated exercise capacity has not been established. When considered collectively, carbohydrate intake during limited recovery appears to primarily determine muscle glycogen resynthesis and repeated exercise capacity. Thus, when the goal is to optimise repeated exercise capacity following short-term recovery, ingesting carbohydrate at an amount of ≥1.2 g kg body mass^−1^·h^−1^ can maximise muscle glycogen repletion. The addition of protein to carbohydrate during post-exercise recovery may be beneficial under circumstances when carbohydrate ingestion is sub-optimal (≤0.8 g kg body mass^−1^·h^−1^) for effective restoration of muscle glycogen and repeated exercise capacity.

## 1. Introduction

Athletes across the myriad range of sports are required to participate in a number of vigorous competitive events interspersed with intense and frequent training sessions and minimal time to recover. The congested annual schedule of many athletes imposes significant loads on their physiological and metabolic systems close to the threshold of exhaustion, from which they are required to recover rapidly in preparation for the subsequent exercise bout [1]. In addition, military personnel also require the capacity to perform prolonged, repeated bouts of physical activity at a given intensity, and habitually active people strive to improve their training regime, and improved recovery following each exercise bout can promote their exercise capacity and adherence to partaking in exercise.

Exercise-induced fatigue is a common sensation that is experienced by any individual undertaking physical exercise. Fatigue during exercise occurs simultaneously at several loci within the neuromuscular system as well as the internal environment [2]. Thus, a multitude of mechanisms have been proposed to explain fatigue, ranging from metabolic disturbances in the motor unit to centrally-mediated perturbations [3,4] and thus—albeit reductionist—fatigue can be broadly characterised as peripheral fatigue and central fatigue [4]. In accordance, research into fatigue is highly complex and a consensus about the aetiology of this phenomenon remains elusive. Not surprisingly, there are numerous definitions of exercise-induced fatigue, as experimentally inducing fatigue is likely to be inherently variable depending the type/duration of exercise and the tools that are used to assess this phenomenon [5]. In the context of this review, however, the term ‘fatigue’ or ‘exhaustion’ are used to denote the inability to sustain running speed at a prescribed intensity, as indicated by the participant. 

To examine the influence of different nutritional interventions on exercise-induced fatigue, exercise capacity protocols often require individuals to exercise until the point of volitional exhaustion (time to exhaustion), while exercise performance entails the completion of a set distance or amount of work as quickly as possible (time trial) [6]. Exercise capacity/performance and recovery from exercise can be enhanced by evidence-based nutritional interventions through the manipulation of different nutritional variables (i.e., nutrient composition, quantity, timing of nutrient ingestion etc.) [7]. For several decades, it has been recognised that carbohydrate availability is critical for exercise capacity [8,9]. This is emphasised during prolonged moderate- to high-intensity exercise where the reliance on endogenous carbohydrate stores becomes increasingly important relative to lower-intensity exercise [10,11]. Studies in humans clearly demonstrate that fatigue during a prolonged exercise bout coincides with low muscle glycogen content [8,10] and the ingestion of carbohydrate is causally related to the maintenance of performance in humans [12] via the attenuation of glycogenolysis [13], the maintenance of euglycaemia, and/or carbohydrate oxidation [9,14]. Therefore, nutritional interventions that increase pre-exercise intramuscular glycogen stores positively correlate with the capacity for exercise, as muscle glycogen depletion closely parallels perception of fatigue [8]. Unsurprisingly, this nutritional modulation of exercise has prompted numerous nutritional interventions to target these fatigue mechanisms, leading to general recommendations to optimise muscle glycogen availability [13,15]. Indeed, both maximising muscle glycogen content prior to exercise and/or sparing its utilisation during exercise can influence endurance capacity [8,16].

There has been less attention with regards to post-exercise nutrition, notwithstanding that recovery is a critical part of training and repletion of muscle glycogen stores is likely to influence the quality of the subsequent exercise bout. Whereas, numerous studies have indicated that muscle glycogen restoration is improved with carbohydrate supplementation (for review see [17]), fewer investigations were instigated to examine muscle glycogen utilisation during a subsequent exercise bout [18,19,20,21,22]. Indeed, the repletion of muscle glycogen content during limited recovery did not translate into improvements in repeated exercise capacity/performance in some circumstances [19,20,23,24], but this is not without contention [21,25]. Although it is well-established that fatigue during prolonged endurance exercise is largely dependent on muscle glycogen concentrations [26,27], other physiological mechanisms, such as central fatigue, liver glycogen depletion, dehydration, and hyperthermia may contribute to the onset of fatigue during endurance-type exercise [28,29,30]

Many studies have been conducted to evaluate the efficacy of mixed-macronutrient supplements, specifically carbohydrate-protein ingestion, given the synergistic effect of these two nutrients on insulin secretion [31,32]. Several cycling-based investigations indicated that increasing carbohydrate intake to recommended ingestion rates is sufficient to maximise glycogen resynthesis and would negate any additional benefit from the inclusion of protein [24,33,34,35,36,37,38]. Nonetheless, other studies using the same exercise modality observed an increase in glycogen resynthesis, indicating a distinct advantage with protein co-ingestion, even when both supplements were matched in their energy content [19,39]. Yet, less is known in relation to the influence of carbohydrate-protein supplementation during short-term recovery upon muscle glycogen repletion and repeated running exercise [20,22,23,40]. It is interesting to note that subsequent endurance capacity has been improved via mechanisms that are independent of glycogen availability when carbohydrate-protein was ingested [40,41], but this is not universal [22]. The exact mechanism behind this ergogenic effect of protein addition to carbohydrate remains to be elucidated and thus our understanding is lacking regarding the plausible mechanistic effects that might justify the inclusion of protein with carbohydrate for optimal short-term recovery from exercise. The current review provides detailed evaluation of the nutritional modulation (carbohydrate and carbohydrate-protein supplementation) of post-exercise glycogen repletion during limited (3–6 h) post-exercise recovery and the influence of these nutrients upon the restoration of exercise capacity following recovery. Unless otherwise stated, the concentrations of muscle glycogen in this review are reported as mmol per kilogram of dry mass per hour (mmol·kg dm^−1^·h^−1^). Thus, data reporting muscle glycogen as mmol per kilogram of wet weight per hour were multiplied by a factor of 4.28 to account for the water weight of the muscle [34].

## 2. Post-Exercise Recovery

A number of factors encompass post-exercise recovery, including rehydration, regeneration and repair of damaged tissue, and restoration of depleted carbohydrate stores [17]. The restoration of endogenous carbohydrate stores is proposed to be crucial in determining the time required for recovery [42]. In contrast to the predominant reliance on carbohydrate metabolism during prolonged exercise, post-exercise recovery is characterised by an accelerated rate of lipid oxidation (≈60% of oxidative metabolism) and “sparing” of carbohydrate oxidation, even under conditions of high-carbohydrate feedings [43,44]. This shift in substrate metabolism clearly demonstrates the high metabolic priority for muscle glycogen resynthesis, whereby lipid oxidation from intra and extra-muscular sources is elevated to meet the fuel requirements [45]. The process of muscle glycogen resynthesis begins immediately following exercise and is most rapid during the first 5–6 h of recovery [46]. Therefore, a view of muscle glycogen resynthesis, the factors that enhance or limit muscle glycogen resynthesis and the nutritional strategies to obtain rapid post-exercise glycogen restoration are discussed.

### 2.1. Glycogen Structure and Localisation

Skeletal muscle glycogen is a highly optimised efficient cellular energy storage system, whereby its branched structure allows for expeditious availability of large amounts of glucose to support the energetic demands of muscular contractions [47]. The biosynthesis of this granule involves three enzymes; glycogenin, glycogen synthase (GS), and branching enzyme [48]. The primer in glycogen synthesis is the protein glycogenin, which incorporates glucose residues through a self-glucosylation reaction and then acts as a substrate for GS and branching enzyme to form two physiologically distinct glycogen pools; proglycogen (low molecular weight acid-insoluble glycogen) and macroglycogen (high molecular weight acid-soluble glycogen) [48,49]. Nevertheless, while it is known that muscle glycogen can be separated into distinct acid-soluble and acid-insoluble fractions [50], the acid-insoluble glycogen does not correspond to proglycogen as both the acid-soluble and acid-insoluble glycogen show similar elusion profiles of high molecular weight glycogen using gel chromatography [51]. The latter finding certainly questions the existence of proglycogen (i.e., low-molecular weight glycogen) as a distinct pool of glycogen, and thus the proglycogen-macroglycogen paradigm may be an artefact of glycogen analysis. 

Apart from its structure, the location of glycogen appears to be an important factor. The majority of investigations utilised acid-base digestion and subsequent enzymatic determination of free glucose for quantification of total muscle glycogen. While this method provides valuable knowledge on glycogen-mediated whole muscle metabolism, this does not allow for an examination of glycogen localisation. More recently, transmission electron microscopy (TEM) has been utilised to determine the subcellular localisation of glycogen within the fibres. This approach has led to the appreciation that skeletal muscle glycogen is distributed across in subsarcolemmal, inter-myofibrillar and intra-myofibrillar pools [52,53] and that resynthesis of glycogen following exercise was initially characterised by an increase in granule number and later by an increase in size [54]. This glycogen heterogeneity likely provides a substrate for specific cellular functions, which is supported when considering the preferential depletion of intra-myofibrillar glycogen during prolonged exercise and the relative distribution of these distinct pools being largely dependent on fibre type, training status, immobilisation and exercise [27]. 

Recent human investigations indicate a close link between localised glycogen depletion and the capacity to perform whole-body exercise. Specifically, a reduction in intra-myofibrillar glycogen levels has been associated with impaired sarcoplasmic reticulum (SR) Ca^2+^ release [52], which was also shown in skinned rat muscle fibres [55]. Furthermore, single mouse muscle fibres exhibited fatigue-induced impairment in tetanic Ca^2+^ release when intra-myofibrillar glycogen were reduced to low levels, suggesting that SR Ca^2+^ release critically depend on energy supply from the intra-myofibrillar glycogen pool [56]. Interestingly, low muscle glycogen following exhaustive exercise was shown to depress muscle SR Ca^2+^ release rate and impair work output following 4 h of recovery in elite endurance athletes [57]. However, when athletes were provided with carbohydrate (1.06 g·kg BM^−1^·h^−1^) during the 4 h recovery period, glycogen concentrations were elevated and SR Ca^2+^ release rate returned to pre-exercise levels, and consequently work output was also normalised. Moreover, an association between glycogen and SR Ca^2+^ release by manipulating muscle glycogen availability during recovery from fatiguing exercise was demonstrated in cross-country skiers [58]. Similar to the study of Gejl et al. [57], the authors demonstrated that carbohydrate ingestion resulted in restoration of muscle glycogen and a normalised in SR Ca^2+^ release, whereas both glycogen and SR Ca^2+^ release were depressed when carbohydrate was withheld from participants [58]. Indeed, a reduced SR Ca^2+^ release will *per se* cause a decrease in tetanic intracellular free Ca^2+^ [53], which is in line with studies demonstrating a faster decrease in tetanic Ca^2+^ in fibres with low muscle glycogen [59,60]. Therefore, the notion that muscle glycogen depletion is associated with disruption in SR Ca^2+^ release and muscular work may lend an explanation to the intimate relationship between the capacity for exercise and muscle glycogen availability. However, the mechanistic evidence to elucidate how and why glycogen levels impair muscle function remain unclear [27,53].

### 2.2. The Two Phases of Muscle Glycogen Resynthesis

It has been indicated that glycogen resynthesis after a bout of exercise occurs in a biphasic pattern [61,62,63]. Initially, there is a rapid increase in glycogen resynthesis at a rate, which occurs independent of insulin concentrations and lasts for 30–60 min post-exercise [42,64]. The presence of this phase is supported by data reporting an accelerated rate of muscle glycogen resynthesis in the initial (0–60 min) post-exercise period, even when insulin was inhibited by somatostatin or when insulin resistant individuals were compared to healthy age-matched controls [62,63]. However, this rate of resynthesis can rapidly decline in the absence of exogenous carbohydrate intake [61,65]. These findings are in agreement with the time-course of the protracted increase in glucose uptake after exercise, whereby a two-fold increase in glucose transporter protein (GLUT)4 translocation can be observed immediately following exercise before gradually declining until reaching pre-exercise levels by 2 h upon cessation [66]. This insulin-independent phase was suggested to only occur when glycogen is depleted to critically low levels (≈150 mmol·kg dm^−1^·h^−1^) at the end of an exercise bout [42,61]. Therefore, it appears that the mechanisms that are responsible for this initial and rapid phase of glycogen restoration involve exercise-induced GLUT4 translocation to the cell membrane and an augmented GS activity secondary to low glycogen concentrations at the end of exercise [42,64].

The second phase of glycogen storage is thought to occur at a substantially lower rate (approximately 80% lower), and is characterised by the affinity of muscle glucose uptake and GS to insulin stimulation [67,68]. This is supported when examining insulin resistant individuals who only show a 3.5% ability to resynthesise muscle glycogen (0.4 mmol·kg dm^−1^·h^−1^) relative to healthy controls [63]. The ingestion of carbohydrate and the associated increase in glucose and insulin concentrations are known to accelerate the rate of muscle glycogen resynthesis during this phase, albeit they remain slower than the insulin-independent phase [65,69]. This greater muscle sensitivity to insulin can persist for longer periods (>48 h) and is reliant on carbohydrate ingestion and the amount of muscle glycogen that has been restored [68,70]. Indeed, a number of factors that are related to the enhanced muscle insulin sensitivity, such as insulin-mediated GLUT4 translocation, increased sensitivity of GS to insulin, muscle glycogen content, and adenosine monophosphate-activated protein kinase (AMPK) activation [64,71,72].

## 3. Nutrient Intake and Muscle Glycogen Resynthesis

Glucose is the precursor for glycogen resynthesis and it is consequently understandable why the amount glucose ingested is such an important determinant of glycogen resynthesis rate [42,73,74]. It has been consistently demonstrated that carbohydrate intake increases glycogen storage above that of water alone [34,75,76]. In the context of recovery from exhaustive exercise, it is known that ingesting 6–12 g carbohydrate·kg^−1^ is sufficient to restore endogenous glycogen reserves when the recovery time is ≥24 h [77,78]. However, athletes and active individuals across a wide range of sporting events train and compete at levels that challenge their daily glycogen stores with minimal time for recovery, with multiple training sessions a day and/or a daily competitive schedule with a metabolic fuel cost that exceeds the endogenous carbohydrate stores. Thus, when the time that is available for recovery is limited (<8 h), neither muscle glycogen nor the capacity for subsequent exercise can be fully restored [17]. It becomes apparent that specific nutritional strategies aimed at acutely accelerating glycogen resynthesis are important considerations in such scenarios. A related but separate question is whether the adaptive response to chronic training is amplified when commencing a number of exercise sessions with low-muscle glycogen concentrations. The reader is referred to a more extensive report on the latter topic elsewhere [79,80].

The optimal carbohydrate feeding strategy to maximise glycogen reserves vary greatly and depend on a number of factors that include, but are not limited to, the amount, timing, and type of the carbohydrate ingested during recovery [17,42]. Therefore, the following sections will discuss these nutritional considerations that are related to glycogen resynthesis during short-term recovery. 

### 3.1. Amount of Carbohydrate Intake

In the absence of carbohydrate ingestion over the post-exercise recovery period, glycogen resynthesis occurs at a rate of ≈2 mmol·kg dm^−1^·h^−1^ [65,81]. Coupled with the wealth of knowledge demonstrating that any ingested carbohydrate of substantial amounts greatly increases muscle glycogen resynthesis than when no carbohydrate is ingested [24,34,57,65,75,76,78] enforces the notion that carbohydrate ingestion is critical for restoration of muscle glycogen. The first study to explore the effects of varying amounts of carbohydrate on muscle glycogen resynthesis during short-term recovery showed that increasing carbohydrate ingestion from 0.18 grams per kilogram of body mass per hour (g·kg BM^−1^·h^−1^) to 0.35 g·kg BM^−1^·h^−1^ concurrently enhances glycogen synthesis rate from 9 to 25 mmol·kg dm^−1^·h^−1^ [82]. When increasing the amount of carbohydrate to 0.70 g·kg BM^−1^·h^−1^, the authors reported no further increase in the glycogen resynthesis rate [82]. While these observations may suggest that the former rate of ingestion would maximise glycogen synthesis, a number of following studies demonstrated that increasing the rate of carbohydrate ingestion from 0.75 to 1 g·kg BM^−1^·h^−1^ elicits a greater glycogen synthetic response than reported previously [83,84,85,86]. Indeed, data from our laboratory indicate that the ingestion of 1.2 g·kg BM^−1^·h^−1^ of carbohydrate during recovery from exhaustive running increases muscle glycogen content when compared to 0.3 g·kg BM^−1^·h^−1^ [21]. It was elegantly demonstrated that carbohydrate ingestion at a rate of 1.2 g·kg BM^−1^·h^−1^ during post-exercise recovery resulted in a 150% greater glycogen synthetic response (from 17 to 45 mmol·kg dm^−1^·h^−1^) relative to a lower dose of 0.8 g·kg BM^−1^·h^−1^ [35]. Because the ingestion of 1.6 g·kg BM^−1^·h^−1^ of carbohydrate seemingly does not further stimulate muscle glycogen resynthesis above that of 1.2 g·kg BM^−1^·h^−1^ [36], the latter may be considered as the optimal amount to maximise muscle glycogen repletion. 

Identifying the precise ‘optimal’ quantity of carbohydrate to maximise glycogen repletion is difficult to ascertain, which may be ascribed to a number of confounding variables, including the type and timing of the ingested carbohydrate, the training status of the participants and the duration of the post-exercise recovery period. More importantly, however, the magnitude of muscle glycogen depletion determines to a large extent its rate of resynthesis [84]. It is therefore notable that the degree of glycogen depletion from a prior exercise bout varies substantially across the literature, such that a range of muscle glycogen concentrations ranging between 25–255 mmol·kg dm^−1^·h^−1^ at the onset of recovery has been reported [18,85]. Together with the known inverse relationship between muscle glycogen content and glucose uptake [87,88,89], the variation in muscle glycogen levels at the onset of recovery are likely to contribute to the large variability in muscle glycogen resynthesis rates between studies. Nonetheless, a positive correlation (*r* = 0.6; *p* < 0.01) exists between the amount of carbohydrate that is ingested during short-term recovery and muscle glycogen resynthesis [17]. Collectively, it is reasonable to suggest that ingesting ≈1.2 g carbohydrate·kg BM^−1^·h^−1^ is likely to maximise muscle glycogen resynthesis and that additional carbohydrate will not further increase this glycogenic response. 

### 3.2. Type of Carbohydrate Intake

A number of studies explored different types of carbohydrate ingestion during short-term recovery in order to establish the most effective means to maximise glycogen storage. An important factor determining the rate of muscle glycogen resynthesis is insulin-mediated glucose uptake by the muscle cells [42]. As such, the elevated insulinaemic response to high as opposed to a low glycaemic index carbohydrate is implicated to accelerate muscle glycogen repletion, at least in the acute (<6 h) recovery phase, with no distinct advantage in longer recovery periods [90]. In contrast, differences in muscle glycogen storage favouring high glycaemic index carbohydrate were shown to persist for up to 24 h [91]. The authors of the latter postulated that these differences may be attributable to the malabsorption of carbohydrate in the low glycaemic index foods, which reinforces the view that the type of carbohydrate ingestion is an important consideration in relation to muscle glycogen resynthesis. Of note, a study by Wee et al. [92] demonstrated that a high-glycaemic index meal ingested 3 h prior to exercise increases muscle glycogen content more than when an isoenergetic low-glycaemic index meal was ingested [92]. While this pre-exercise meal may be extrapolated to reflect a recovery meal prior to subsequent exercise, the data from this study must be interpreted with caution, as the metabolic perturbations to a repeated exercise bout remain largely unknown and may differ from a prior exercise bout. 

Fructose stimulates modest amounts of insulin stimulation relative to glucose and sucrose, mainly ascribed to its preferential hepatic glycogen resynthesis [93]. As a consequence, fructose ingestion does not stimulate muscle glycogen resynthesis to the same magnitude as when glucose or sucrose are ingested [94,95], which is also supported by carbon-13 nuclear magnetic resonance (^13^C-NMR) data [96]. Furthermore, it appears that sucrose and glucose stimulate muscle glycogen resynthesis at a similar magnitude [82]. More recently, it was demonstrated that when ≥1.2 g carbohydrate·kg BM^−1^·h^−1^ are ingested during recovery from an initial exhaustive bout, glucose, and glucose + fructose, or glucose + sucrose ingestion elicit similar muscle glycogen resynthesis rates [97,98]. Interestingly, these responses are commonly observed in the presence of variable insulinaemia [99,100], bringing into question the importance of subtle differences in insulinaemia when large amounts of carbohydrate are ingested as fructose plus glucose, compared to glucose only. It would therefore be prudent to ingest a mixture of glucose and fructose to provide an optimal dose of carbohydrate for the effective restoration of both muscle and liver glycogen stores [25,82,97] and reduce gastrointestinal distress when ingesting large amounts of carbohydrate [98]. Sucrose contains equimolar amounts of glucose and fructose, rendering this disaccharide favourable in optimising overall endogenous carbohydrate reserves (liver and muscle glycogen), both of which have been shown to associate with the capacity for exercise [25]. Interestingly, sucrose ingestion (1.5 g·kg BM^−1^·h^−1^) was shown to accelerate liver glycogen content during 5 h of recovery following glycogen-depleting exercise without any gastro-intestinal complaints when compared to an isocaloric glucose solution [99]. Furthermore, recent data demonstrate that the ingestion of glucose + fructose restores endurance capacity following short-term recovery more effectively than when the same amount of glucose were ingested [101].

Manipulation of the form (solid vs. liquid) of carbohydrate ingestion does not seem to influence the rate of muscle glycogen resynthesis during recovery [69,83,102]. These observations are in concert of the view that muscle glycogen resynthesis is unlikely to be limited by gastric emptying [102]. Rather, a combination of other factors, such as the amount of carbohydrate, intestinal absorption and delivery into circulation, extraction of glucose by other tissues, and the capacity for glucose transport into the muscle [42]. In relation to this contention, it is interesting to note that the intestinal absorption of fructose occurs though a different transporter (sodium-independent glucose transporter protein 5; GLUT-5) than glucose (sodium-dependent glucose transporter 1: SGLT1) and thus the combined ingestion of glucose + fructose or glucose + sucrose accelerate overall carbohydrate delivery, which may be an important consideration during acute post-exercise recovery [98], particularly when large amounts (≥60 g·h^−1^) of glucose are ingested and thus saturating SGLT1 [103,104].

### 3.3. Timing of Carbohydrate Intake

Skeletal muscle blood flow and sensitivity to nutrient provision are thought to be augmented following a prior exercise bout that substantially depletes muscle glycogen, which emphasises the influence of timing of carbohydrate provision during the post-exercise recovery [105]. In fact, the rate of muscle glycogen resynthesis was shown to be ≈25 mmol·kg dm^−1^·h^−1^ over 4 h of recovery when carbohydrate was provided immediately following exercise [65]. When carbohydrate provision was delayed by only 2 h, a considerable reduction to 14 mmol·kg dm^−1^·h^−1^ in the rate of muscle glycogen resynthesis was reported [65]. In concurrence, delaying the ingestion of a mixed macronutrient recovery beverage by 3 h was shown to reduce net leg glucose uptake by 65% during recovery when compared to immediate ingestion [106]. These findings may arguably be expected given that insulin sensitivity and the capacity for glucose uptake are the most rapid in the initial few hours of recovery, and when carbohydrate is withheld during this period rapid reversal of these effects can be observed [66,68,107]. Nevertheless, a study by Parkin and colleagues [108] sought to determine the effects of delayed carbohydrate feeding of 2 h on the recovery of muscle glycogen over 8 h of recovery and reported no differences in muscle glycogen resynthesis rates [108]. While these findings may appear contradictory, it remains possible that glycogen resynthesis rates were higher in the study of Parkin et al. [108] over the initial 4 h of recovery, with the reversal of this augmented glycogen synthetic rate later in recovery secondary to the well-established and tightly controlled inverse relationship between muscle glycogen content and GS activity [109]. Therefore, it would not be unreasonable to postulate that the effect of timing of carbohydrate on muscle glycogen resynthesis is magnified when the recovery time is shorter. Irrespective of the progressive insulin resistance that occurs later in recovery, it would be logical consume carbohydrate as soon as practically possible to initiate the effective time for muscle glycogen resynthesis. 

Similar to the effects of the glycaemic index of foods during longer recovery periods (i.e., 24 h), the frequency of carbohydrate intake does not appear to influence overall muscle glycogen resynthesis [110,111]. However, when the recovery time is limited, the frequency at which the carbohydrate is ingested may have an influence. Specifically, those studies that adopted a feeding regimen at 2 h intervals typically reported muscle glycogen resynthesis rates between 14–25 mmol·kg dm^−1^·h^−1^ [65,82,102]. It may therefore be argued that the aforementioned frequency may not be a sufficient nutritional strategy to maintain the elevation in insulin and consequently maximise the activation of GLUT-4 and GS [112]. On the other hand, when carbohydrate feeding occurs within 15–30 min intervals, the muscle glycogen resynthesis rate has been found to be approximately 40% higher than when supplementing every 2 h [33,34,35,97,113]. It should be recognised, however, that there is currently no study that directly examined the frequency of supplementation on the rate of muscle glycogen storage. Nonetheless, it is reasonable to suggest from the studies cited above that when a more rapid glycogen restoration is required during short-term recovery, a more frequent feeding pattern may be favourable to achieve this target. 

### 3.4. Protein Co-Ingestion with Carbohydrate

While it is known that glucose is a major stimulus for pancreatic insulin secretion, insulin concentrations are stimulated when healthy individuals ingest protein or receive a mixture of amino acids intravenously [114]. Moreover, amino acids act synergistically when co-ingested with carbohydrate to potentiate insulin secretion [31,32,115]. Oral ingestion of an amino acid mixture, particularly with sufficient amounts of leucine and phenylalanine, produces strong insulinotropic effect when compared with carbohydrate only solutions [32]. The insulinotropic effects of protein ingestion are due to the dual-action of stimulating incretin hormone secretion by the enteroendocrine cells of the intestine, in addition to direct stimulate of the pancreatic beta-cells by amino acid concentrations [116,117]. The incretin hormones would potentiate insulin secretion under conditions of elevated glycaemia, and thus may play an important role in glycogen resynthesis with protein-carbohydrate co-ingestion [117]. It was also demonstrated that a hydrolysed protein fraction provides a distinct advantage in stimulating insulin release over its intact form, being mainly related to an accelerated rate of digestion and absorption and the resultant relative hyperaminoacidaemia of the former [118,119]. Furthermore, whey protein is a greater insulin secretagogue than casein [120], presumably associated with the greater leucine content combined with its rapid plasma amino acid availability.

In addition to the findings regarding the most effective amino acid/protein fraction cited above, a further point related to the amount of protein to effectively stimulate insulin warrants discussion. It was previously suggested that increasing the amount of protein in a carbohydrate-protein mixture from 0.2 to 0.4 g·kg BM^−1^·h^−1^ may be associated with greater insulin response, albeit these differences were only significant when reported as an incremental area under the curve [121]. A possible explanation would be that the latter study provided wheat protein as opposed to whey protein, which contains substantially higher leucine content than the former. These differences may have considerable implications as leucine is a known modulator of insulin signaling [122]. This was supported by the same study, whereby free leucine and phenylalanine were added to the wheat protein fraction, and consequently increased insulin concentration relative to a carbohydrate-only beverage [121]. More recently, a dose-dependent relationship between the amount of whey protein co-ingested during recovery and insulin secretion was found when 0.1 as compared to 0.3 g·kg BM^−1^·h^−1^ were ingested [123]. When considered collectively, the available evidence that reported insulinotropic properties with carbohydrate-protein ingestion have included 0.3–0.4 g·kg BM^−1^·h^−1^ of protein [20,22,33,34,37,124]. In contrast, protein was provided at a lower dose in those studies that showed no effect of protein co-ingestion on insulin secretion relative to carbohydrate-only [38,39,75,76]. 

The addition of whey protein hydrolysate (with or without additional free essential amino acids) to a carbohydrate supplement is known to result in a greater insulin response [32,120,125]. Concurrently, adding protein to carbohydrate following recovery was reported to accelerate the rate of muscle glycogen resynthesis relative to a carbohydrate-only supplement when ingested in moderate amounts (i.e., ≤0.8 g carbohydrate·kg BM^−1^·h^−1^) [19,35,39,124,126,127,128]. While three of the above cited investigations matched the carbohydrate content between supplements, it is difficult to conclude whether the addition of protein or the higher caloric intake was related to the enhanced glycogen resynthesis rate [124,127,128]. In two investigations, however, muscle glycogen resynthesis was augmented when an isoenergetic carbohydrate-protein supplement was provided [19,39]. Notably, muscle glycogen resynthesis was accelerated in the study of Ivy et al. [39], irrespective of the fact that insulin concentrations during recovery were similar between supplements. Coupled with the fact that insulin concentrations were not reported in the other investigation [19], whether the enhanced glycogenic effect in those investigations was related to hyperinsulinaemia is questionable. Van Loon et al. [35] compared a carbohydrate-protein mixture (0.8 g carbohydrate·kg BM^−1^·h^−1^ plus 0.4 g protein·kg BM^−1^·h^−1^) against both a carbohydrate-matched (0.8 g carbohydrate·kg BM^−1^·h^−1^) and an energy-matched (1.2 g carbohydrate·kg BM^−1^·h^−1^) carbohydrate supplement. The authors demonstrated that the addition of protein effectively increased the insulin concentrations and glycogen storage by twofold when compared to the carbohydrate-matched supplement. However, replacing the protein fraction by additional energy in the form of carbohydrate achieved similar results, with no differences in glycogen resynthesis between the carbohydrate-protein mixture and the isoenergetic carbohydrate supplement [35]. The latter findings clearly demonstrate that carbohydrate intake should be greater than 0.8 g carbohydrate·kg BM^−1^·h^−1^ to allow for maximal glycogen resynthesis rates. 

**Figure 1:**
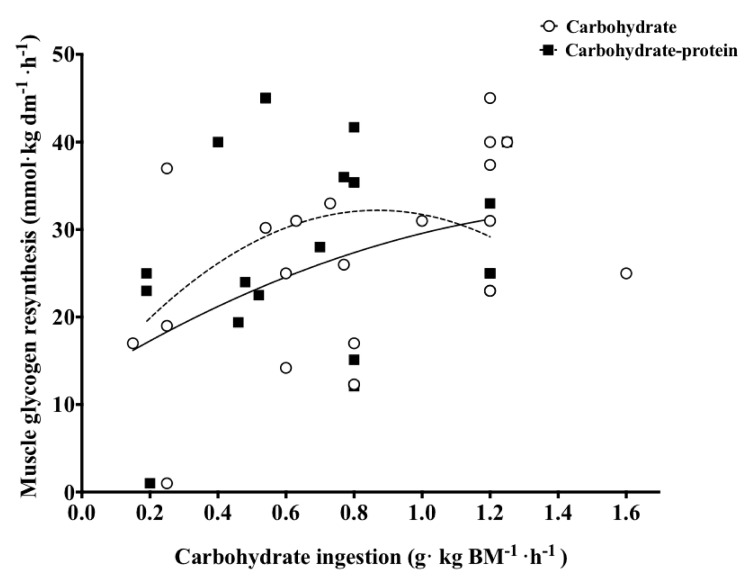
Reported rates of muscle glycogen resynthesis across 18 different investigations that have measured muscle glycogen concentrations during short-term (2–6 h) recovery with varied rates of carbohydrate with or without protein in humans [19,20,22,24,33,34,35,36,37,38,39,40,75,76,124,126,130,132]. The trend lines denote the suggested patterns of muscle glycogen resynthesis with each treatment (solid trend line represents carbohydrate ingestion while broken trend lines represent carbohydrate-protein ingestion).

Equally, a number of other investigations proposed that the addition of protein does not further increase the rate of muscle glycogen resynthesis, despite a higher insulinaemic response [20,22,33,34,37,129,130,131,132]. Noticeably, five of the studies cited previously examined whether the rate of muscle glycogen resynthesis by ingesting the proposed ‘optimal’ dose of carbohydrate (1.2 g·kg BM^−1^·h^−1^) can be exceeded with the addition of protein/amino acids, and none reported an accelerated rate of muscle glycogen resynthesis with protein co-ingestion [33,34,37,130,131]. An important distinction between the studies showing a glycogenic effect of protein co-ingestion and those with contradictory findings may be related to the precise amount of carbohydrate ingested during post-exercise recovery. When compiling the available data in humans (Figure 1), it becomes apparent that the ingestion of ≈1.2 g carbohydrate·kg BM^−1^·h^−1^ is likely to maximise muscle glycogen resynthesis rate and further stimulating insulin with the addition of protein does not appear to influence glycogen storage [22,33,34,37,130,131]. In contrast, when ≤0.8 g carbohydrate·kg BM^−1^·h^−1^ is ingested, the addition of protein with this relatively moderate amount of carbohydrate may enhance muscle glycogen resynthesis rates [19,35,39,124,126].

It should be noted there are some inconsistent reports in the literature. For example, providing a carbohydrate dose of ≤0.8 g carbohydrate·kg BM^−1^·h^−1^ with protein was shown to be ineffective in augmenting muscle glycogen relative to an energy-matched [40,75,76,129] or carbohydrate-matched supplement [20]. Although these discrepancies are not fully clear, they may be related to differences in quantifying muscle glycogen, the provision of sub-optimal amounts of protein to stimulate insulin secretion, or the specific type of exercise that was performed prior to recovery. Indeed, the study of Rotman et al. [129] used ^13^C-magnetic resonance spectroscopy to quantify glycogen, and while this method has been validated and shows a high correlation (*r* = 0.95; *p* < 0.001) with the needle biopsy technique [133], determining the rate of resynthesis is limited due to the absence of absolute glycogen concentrations. Additionally, the studies of Betts et al. [20] and Lunn et al. [40] employed an exercise protocol (i.e., running) that was not commonly used in the other cycling-based investigations. Indeed, it has been demonstrated that performing muscular contractions with an eccentric component, such as running impairs both contraction- [134] and insulin-induced glucose uptake by the muscle [135]. As such, eccentric exercise has been shown to impair muscle glycogen resynthesis [136], which was corroborated by the study of Betts et al. [20] as reflected by the relatively low rates of glycogen resynthesis in recovery (≈12 mmol·kg dm^−1^·h^−1^). Of note, only one study examined the role of an exhaustive prior running exercise bout on muscle glycogen resynthesis [22], which demonstrated near maximal glycogen reynthesis rates (≈40 mmol·kg dm^−1^·h^−1^). This may be an important distinction, given that the magnitude of glycogen depletion influences the rate of muscle glycogen restoration during post-exercise recovery [84]. 

**Figure 2:**
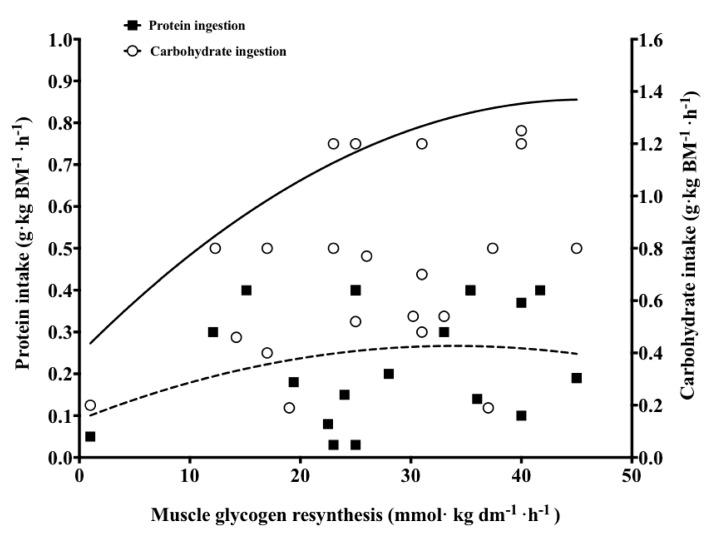
Reported rates of muscle glycogen resynthesis across 18 different investigations that have measured muscle glycogen concentrations during short-term (2–6 h) recovery with varied amounts of protein added to carbohydrates in humans [19,20,22,24,33,34,35,36,37,38,39,40,75,76,124,126,130,132]. The trend lines denote suggested carbohydrate intake (solid trend line) and protein intake (broken trend line) upon muscle glycogen resynthesis.

As mentioned previously, the addition of at least 0.3–0.4 g·kg BM^−1^·h^−1^ protein may be required to achieve the synergistic effect of a carbohydrate-protein mixture on insulin secretion. In accordance, neither muscle glycogen storage nor insulin were significantly elevated when protein was included (≤0.2 g·kg BM^−1^·h^−1^) in a carbohydrate-protein mixture relative to carbohydrate alone [38,40,75,76]. These findings may help explain the lack of effect of protein co-ingestion when added to relatively moderate amounts of carbohydrate. However, an accelerated rate of muscle glycogen resynthesis has been reported when modest amounts of protein (<0.2 g·kg BM^−1^·h^−1^) were added to ≈0.6–0.7 g carbohydrate·kg BM^−1^·h^−1^ [19,39], despite the fact that no significantly increased insulin concentration was observed in the carbohydrate-protein treatment [39]. This led authors of the latter study to speculate that alternative mechanisms may exist in relation to an accelerated muscle glycogen synthetic response. Unfortunately, the source of protein was not reported in that investigation, however, essential amino acids, such as isoleucine and leucine, may act in concert to facilitate glucose uptake and consequent incorporation into muscle glycogen independent of insulin [137,138], presumably by an increased phosphorylation of Akt substrate of 160kDa (AS160) signaling in the absence of insulin [139]. Collectively, if carbohydrate ingestion is ≤0.8 g·kg BM^−1^·h^−1^, it appears that 0.3–0.4 g·kg BM^−1^·h^−1^ of protein should be co-ingested to maximise muscle glycogen resynthesis during short-term recovery (Figure 2). 

The fate of glucose following carbohydrate-protein ingestion must be considered to assess the relevance of hyperinsulinaemia during post-exercise recovery on glucose disposal and subsequent incorporation as endogenous carbohydrate. In this regard, it is interesting to note that a significant inverse relationship (*r* = 0.99; *p* < 0.001) exists between the amount of protein intake and blood glucose concentration [140]. In concurrence, the majority of investigations on post-exercise protein co-ingestion have reported lower blood glucose concentrations relative to a carbohydrate-only beverage [24,34,35,38,124,131]. Nevertheless, whether this attenuated glycaemic response was due to a delayed glucose appearance into circulation or an increased glucose uptake by the muscle remains debatable. Although it is tempting to speculate that the lower glucose levels with protein co-ingestion are reflective of an increased peripheral glucose uptake secondary to insulin stimulation, there is some evidence to refute this contention in relation to muscle [131] and liver [130] glycogen repletion. Thus, while the addition of protein to carbohydrate resulted in a ≈100–190% higher insulin and ≈35–42% lower glucose response, the rate of glucose disappearance using continuous glucose tracer infusions was identical between a carbohydrate control and the carbohydrate-protein mixtures [125]. The authors, however, noted a 12% reduction in glucose appearance, implying the lower glucose response with carbohydrate-protein mixtures may partly involve a delayed appearance of glucose into the systemic circulation. Nevertheless, studies in rodents have demonstrated a hypoglycaemic effect of certain amino acids and the consequent increased glucose uptake by the muscle [137,138,139]. While the hypoglycaemic effect of a mixture of amino acids has been reported in humans, whether this is related to an increased glucose uptake remains unclear [131]. A number of possible mechanisms may therefore be attributed to the relatively delayed appearance of glucose in the latter study, and while a slower gastric emptying and/or intestinal absorption may contribute to the delayed glucose appearance, it is unlikely to fully explain to lower glucose response following carbohydrate-protein ingestion [125]. An alternative explanation would be an insulin-induced suppression of hepatic glucose output, which is known to inhibit gluconeogenesis and glycogenolysis by ≈55 and 100% when insulin is stimulated to approximately 450 pmol/L, respectively [141]. Indeed, insulin concentration in the study of Kaastra et al. [125] reached 480–700 pmol/L, and would therefore be expected to exert an inhibitory effect of hepatic glucose output. 

## 4. Restoration of Exercise Capacity Following Short-Term Recovery

Given the intrinsic link between muscle glycogen depletion and endurance capacity, restoration of these endogenous carbohydrate stores is central to the recovery process [23,142]. While performance decrements and the declined ability to maintain repeated intensified training may be the outcomes of insufficient glycogen repletion between exercise bouts during long-term recovery (i.e., ≥24 h) [143,144,145] and that nutrition is inherently associated to this process, little is known regarding the optimal nutritional intervention that could translate into an enhancement in subsequent exercise capacity following short-term recovery (Table 1). For example, subsequent endurance capacity (60–70% VO_2max_) can be improved when ≈0.3–0.7 g·kg^−1^·h^−1^ of carbohydrate is ingested during short-term recovery when compared to a placebo fluid [146,147,148]. On the other hand, other studies found no effect of carbohydrate ingestion on the subsequent cycling endurance capacity [25], intermittent running endurance capacity [149] or cycling time trial performance [24] when compared to a placebo beverage ingested during preceding recovery period. Some of these paradoxical findings may be related to subtle differences in the adopted experimental protocols, such as measuring endurance capacity under warm environmental conditions [147,148], which could trigger a more central mechanism to the onset of fatigue independent of substrate depletion [150]. Additionally, differences in feeding frequency may also contribute to the disparity between the studies through frequent [23,151], less frequent [24,146,149,152], or single bolus [25] provisions of carbohydrate during the imposed recovery period. Nonetheless, there is evidence to suggest that the frequency of carbohydrate intake during short-term recovery does not influence subsequent endurance capacity [153]. Indeed, the ambiguity of the efficacy of ingesting carbohydrate on subsequent endurance performance is present irrespective of the frequency of ingestion. 

It can therefore be postulated that the ingestion of carbohydrate can enhance endurance capacity relative to a placebo. However, increasing the amount of carbohydrate during limited recovery may not yield further improvements in subsequent endurance capacity [152,154], notwithstanding that a dose-dependent effect was reported in a later study [23]. Regardless of the fact that the exercise protocol was similar between those studies, the characteristics of participants were profoundly different. Specifically, lower blood lactate and higher VO_2max_ values were observed in the study of Betts and colleagues [23] when compared to the other investigations [152,154], indicative of a more aerobically-trained sample in the former. Thus, training status may further explain the mixed results regarding endurance capacity following provisions of different amounts of carbohydrate, given that well-trained individuals who are familiarised with exercise capacity testing exhibit a more reliable reflection on performance measures [155].

Another possible explanation for the discrepant findings between the study of Betts et al. [23] and those that did not observe a dose-dependent effect on repeated exercise capacity [152,154] may be related to the precise amount of carbohydrate that was ingested during recovery. It was shown that increasing carbohydrate during recovery from 0.15 to 0.53 g·kg BM^−1^·h^−1^ did not elicit an improvement in the capacity to run to exhaustion at 70% VO_2max_ [154]. These similar conditions were subsequently investigated by Tsintzas et al. [18] to assess glycogen storage during recovery and its subsequent utilisation during a second bout. Although net muscle glycogen resynthesis rates were ≈250% greater when carbohydrate was ingested at a rate of 0.53 g·kg BM^−1^·h^−1^, glycogen utilisation during subsequent exercise was not different between treatments [18]. These findings may suggest that glycogen content may not be the most important factor in restoring endurance capacity when the recovery period is limited. It should be recognised, however, that the second bout did not measure endurance capacity (i.e., the second run was a fixed duration of only 15 min), and that glycogen utilisation rates towards the end of an exhaustive bout may have differed between the trials. Furthermore, the amount provided in the latter study was much lower than the amount of carbohydrate suggested to maximise post-exercise glycogen resynthesis rates of ≈1.2 g·kg BM^−1^·h^−1^ [35,36]. Thus, when ingesting carbohydrate at a rate that approaches the aforementioned recommended carbohydrate intakes to maximise muscle glycogen stores, an enhancement in endurance capacity was observed relative to modest lower amounts (0.8 vs. 1.1 g·kg BM^−1^·h^−1^) of carbohydrate [23]. This may imply that increasing carbohydrate ingestion following a prior exercise bout is likely to increase muscle glycogen resynthesis during limited recovery, which, in turn, would result in an improvement in repeated exercise capacity. To address this, we have recently reported that higher intakes of carbohydrate (1.2 g·kg BM^−1^·h^−1^) during recovery from exhaustive running substantially increase muscle glycogen content before the start of subsequent exercise when compared to the ingestion of modest amounts (0.3 g·kg BM^−1^·h^−1^) of carbohydrate [21]. Interestingly, the restoration of exercise capacity was ≈65% greater in the high carbohydrate treatment. Coupled with the fact that fatigue coincided with similar critically low levels of muscle glycogen (≈75 mmol·kg dm^−1^), it was therefore concluded that availability of skeletal muscle glycogen is an important factor in the restoration of endurance capacity following short-term recovery [21]. 

The addition of protein to a carbohydrate supplement may accelerate the rate of muscle glycogen resynthesis [19,39,124]. It would therefore be reasonable to suggest that protein-co-ingestion has the potential to improve subsequent endurance capacity, given the relationship between pre-exercise muscle content glycogen and exercise time to exhaustion [8]. In this regard, the restoration of muscle glycogen during limited recovery is considered a possible mechanism for the ostensible ergogenic effect of carbohydrate-protein ingestion on repeated exercise, and thus glycogen restoration will only be discussed in relevance to subsequent endurance capacity in this section (Table 2). Moreover, the interaction of ingested amino acids with the liver may also be relevant for short-term recovery, as liver glycogen resynthesis appears to be an important factor affecting subsequent exercise. Some support for this notion can be obtained when considering the correlation between the recovery of exercise capacity and the restoration of bodily endogenous carbohydrate stores (muscle and liver glycogen; *r* = 0.55; *p* < 0.05) relative to restoration of hepatic glycogen (*r* = 0.53; *p* < 0.05) [25] or muscle glycogen (*r =* 0.45; *p* < 0.05) [21] stores alone. However, a paucity of information exists in relation to the effects of protein co-ingestion on liver glycogen metabolism and/or repeated exercise capacity. 

In fact, very few studies directly measured the rate of glycogen resynthesis during the recovery phase and the subsequent endurance capacity [40,126] or performance [24] with protein co-ingestion. Notwithstanding that the study of Williams and colleagues [126] employed different cohorts to separately examine the role of carbohydrate-protein on glycogen resynthesis and subsequent endurance capacity, the authors showed improvement in cycling time to exhaustion at 85% VO_2max_ when protein was added to a carbohydrate relative to a carbohydrate-only supplement (31.1 ± 3.2 and 20.0 ± 2.0 min, respectively). Nonetheless, the experimental design of that study failed to demonstrate whether the improvements were attributed to the protein fraction *per se* or the 167% increase in carbohydrate intake, or indeed therefore the 233% increase in caloric intake; an important factor in determining the rate of muscle glycogen resynthesis during post-exercise recovery [76]. The provision of these two supplements at a similar rate of ingestion were investigated to determine the restoration of exercise capacity and reported that cycling capacity may actually be impaired with the inclusion of protein [158], albeit a milk-based carbohydrate-protein mixture did not show these negative effects [41]. 

Regardless of these limitations, the findings of Williams et al. [126] provide intriguing evidence that repeated exercise capacity may be enhanced with the presence of protein or with increasing energy intake in a dose-dependent manner. A more recent investigation accounted for the caloric equivalency when comparing a carbohydrate-protein as opposed to an isocaloric carbohydrate beverage on recovery rates and repeated exercise capacity [40]. Although no differences were noted in muscle glycogen resynthesis during 3 h of recovery, subsequent endurance capacity was significantly improved with the ingestion of the carbohydrate-protein mixture [40]. It is noteworthy that the beneficial outcomes for protein intake in this study cannot be solely attributed to the protein fraction, as the study used chocolate milk that includes other nutrients that may affect glycogen storage and/or subsequent performance, such as caffeine [160]. Furthermore, the study utilised a capacity test that induced fatigue within ≈3 min that may suggest that factors other than glycogen-dependent mechanisms were responsible for the postponed termination of exercise [157]. 

Another study of relevance when examining repeated exercise following limited recovery is the study by Ferguson-Stegall et al. [24] In concurrence with the many of the studies in the literature, when supplements were matched for energy content and provided in optimal amounts (i.e., ≥1 g·kg BM^−1^·h^−1^), protein did not appear to augment glycogen resynthesis beyond ingesting carbohydrate [24]. Of note, the aforementioned study did not report absolute glycogen concentrations during recovery and hence limits the interpretation of these data. Notwithstanding this evidence, repeated cycling performance was shown to improve beyond that of an isocaloric carbohydrate following the ingestion of a milk-based carbohydrate-protein mixture [24], lending support to the notion that improvements in subsequent exercise may be unrelated to muscle glycogen resynthesis during short-term recovery. 

Further studies investigated the efficacy of protein feeding during the limited recovery period on subsequent endurance capacity [23,156,157] and performance [19,123,159,161] without the assessment of glycogen concentrations following an initial exercise bout. The findings of these investigations are inconsistent with some showing ergogenic effects of acute carbohydrate-protein feeding of both the capacity to sustain endurance exercise [23] and performance [161], while others did not reach similar conclusions [19,123,156,157,159]. Similar to the nutritional considerations regarding muscle glycogen resynthesis, the precise amount of ingested carbohydrate and whether the supplements were matched for energy content may provide a possible explanation for these discrepant findings. For example, the study by Betts et al. [23] demonstrated that the addition of protein (0.3 g·kg BM^−1^·h^−1^) to a carbohydrate supplement (0.8 g·kg BM^−1^·h^−1^) restored the capacity for repeated exercise more completely than when a carbohydrate-matched supplement was ingested. However, recovery of exercise capacity was restored to a similar magnitude in the carbohydrate-protein mixture when compared with an isocaloric carbohydrate supplement [23]. 

These findings clearly demonstrate that the addition of protein can enhance repeated exercise capacity when increasing the caloric content of a carbohydrate supplement, and that carbohydrate intake should be ≥1.1 g carbohydrate·kg BM^−1^·h^−1^ to allow for a greater restoration of exercise capacity. Interestingly, these identical nutritional provisions were reported in a subsequent study by the same authors and reported no acceleration of muscle glycogen resynthesis between a carbohydrate-protein mixture and a control solution of matched carbohydrate content [20]. This provides further indication that an enhancement in repeated exercise can occur with carbohydrate protein ingestion independent of accelerated muscle glycogen resynthesis. Rather, a consistent finding was an increased rate of whole-body carbohydrate oxidation and maintenance of euglycaemia during the second bout after carbohydrate-protein ingestion [20,23]. Coupled with the fact that glycogen degradation was similar between a carbohydrate-matched control beverage and carbohydrate-protein mixture [20], it is reasonable to suggest that an improved maintenance of euglycaemia, and/or therefore increased extra-muscular carbohydrate oxidation may explain, at least in part, the ergogenic effect of protein co-ingestion during recovery. 

It was previously proposed that the addition of protein may provide precursors for the *de novo* synthesis of tricarboxylic acid cycle intermediates and thus may enable anaplerotic replenishment of tricarboxylic acid cycle flux in the skeletal muscle [162]. While a decline in tricarboxylic acid cycle intermediate pool was shown during prolonged exercise, aerobic provision was not compromised, as evidenced by stable limb oxygen uptake during exercise and no change in muscle phosphocreatine concentration, which is a sensitive indicator of mitochondrial respiration [163]. It was therefore concluded from the latter study that changes in muscle tricarboxylic acid cycle intermediates are not causally related to the capacity for aerobic energy provision during prolonged exercise. 

Another proposed mechanism for the ergogenic effect of protein co-ingestion may be related to the role of amino acids in brain function and postponement of central fatigue [164]. Although there is some evidence to suggest that the ingestion of protein or amino acids reduces perceptions of fatigue during exercise [165,166], it remains debatable whether the inclusion of protein with carbohydrate can improve exercise performance through attenuated sensation of fatigue [167,168]. Interestingly, a recent study in rodents reported that the co-ingestion of protein with carbohydrate attenuates skeletal muscle glycogen depletion during exercise [169]. The latter study demonstrated that pre-exercise ingestion of glucose plus whey protein hydrolysate caused an attenuation in muscle glycogen depletion during a subsequent exercise, which was concomitant with an activation of key enzymes that regulate glucose uptake and glycogen synthesis (Protein kinase B (Akt), Protein kinase C and glycogen synthase) during exercise relative to water ingestion [169]. Thus, the possibility of protein to attenuate glycogen degradation or to increase the net balance of glycogen metabolism (an increase in the ratio of glycogen synthesis and degradation) may be a candidate mechanism for the ergogenic effects of protein co-ingestion. Whether this is partly due to protein providing an additional fuel for oxidation, either directly or indirectly via gluconeogenesis, remains to be determined. Recent evidence in humans, however, has shown that the ingestion of carbohydrate-protein solution during short-term recovery did not affect glycogen metabolism nor mediate an improvement repeated exercise capacity more than an isocaloric carbohydrate solution [22]. 

## 5. Summary

Optimising short-term recovery is an important consideration for both athletes who train and compete with limited time to recover and recreational exercisers who would benefit from the avoidance of residual fatigue, which could negatively influence their sustained participation in physical activity. The notion that muscle glycogen is central to recovery is based on the plethora of experiments demonstrating a causal relationship between muscle glycogen depletion during an initial prolonged exercise and the onset of fatigue. Thus, previous research focused on the effects on different nutritional interventions to increase the availability of this substrate, albeit the precise nutrient amount/composition remains debatable. 

**Figure 3:**
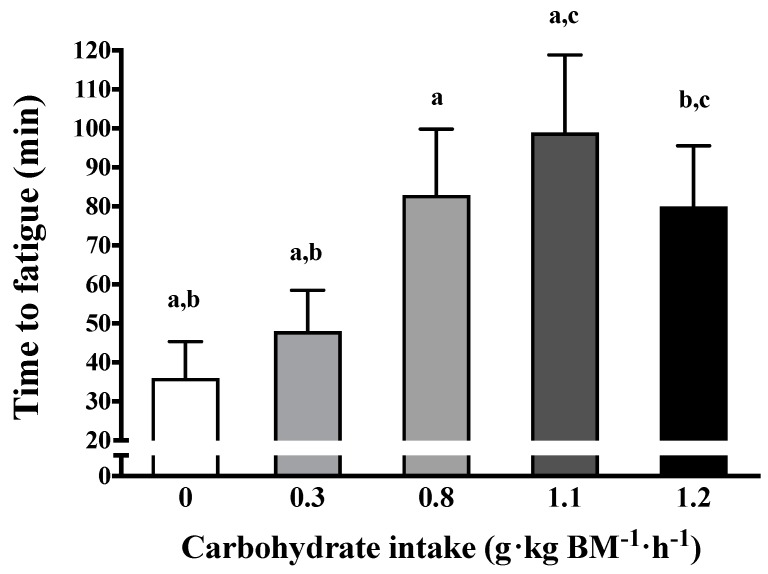
Reported carbohydrate intakes during 4 h recovery and repeated running [21,22,23]. Values are mean ± SD. Values with similar lower cases are different (*p* < 0.05).

The present review examined the role of carbohydrate and protein ingestion from an initial exercise bout on muscle glycogen metabolism during short-term recovery and subsequent exercise capacity. Collectively, the findings of this review support the notion that repeated exercise capacity following short-term recovery is influenced by carbohydrate ingestion, which is consistent with the role of carbohydrate supplementation on an initial prolonged exercise bout [8,16]. A dose-dependent relationship between carbohydrate ingestion during short-term recovery and the restoration of endurance capacity may be present (Figure 3), but further research is warranted regarding the precise dose, type, and/or frequency of carbohydrate feeding during limited recovery to optimise repeated exercise capacity.

The co-ingestion of protein may further accelerate muscle glycogen content relative to carbohydrate alone under circumstances when the carbohydrate-protein solution provides more calories than a carbohydrate-only solution and/or when carbohydrate ingestion during short-term recovery from prolonged exhaustive exercise is suboptimal (i.e., ≤0.8 g·kg BM^−1^·h^−1^). Substituting a fraction of the carbohydrate with protein at an amount of 0.3–0.4 g·kg BM^−1^·h^−1^ during short-term recovery promotes muscle glycogen repletion at the same rate as an energy-matched carbohydrate at the recommended (≈1.2 g·kg BM^−1^·h^−1^) ingestion rates [22]. Overall, the addition of protein at an amount ≥0.1 g·kg BM^−1^·h^−1^ appears to mediate an ergogenic benefit upon the restoration of endurance capacity (Table 2) when this adds to the total energy intake relative to a carbohydrate control or when carbohydrate intake is suboptimal (≤0.8 g·kg BM^−1^·h^−1^). Thus, it may be that energy intake *per se* and not macronutrient composition during recovery influence repeated exercise capacity. 

## 6. Future Directions

Muscle glycogen availability was implicated as a major determinant of fatigue during a repeated exercise bout. In this regard, in determining the role of liver glycogen availability prior to a second exhaustive running bout would be central to further understanding fatigue mechanisms during repeated exercise, particularly given the importance of liver glycogen in restoration of endurance capacity in cycling [25]. Interestingly, we found that repeated exercise capacity was enhanced by 12 min when carbohydrate was ingested during post-exercise recovery in amounts that were sufficient only to satisfy hepatic glycogen sequestration relative to water ingestion [21]. This certainly argues the possibility that liver glycogen availability may be an important factor in relation to the onset of fatigue during a repeated exhaustive running bout, which is possible to quantify using ^13^C-magnetic resonance spectroscopy. 

The rate of carbohydrate intake during recovery is an important determinant of muscle glycogen resynthesis [17,77]. Surprisingly, however, few investigations directly assessed the relative dose-response of carbohydrate ingestion (and indeed feeding frequency) on the restoration of muscle glycogen. Rather, the general recommendations on carbohydrate ingestion during short-term recovery are based on comparisons of the rate of glycogen resynthesis relative to the carbohydrate intake across multiple studies. In relation to the latter, exploring varied carbohydrate intakes during limited recovery on restoration of exercise capacity would help to further clarify the presence of a dose-response relationship between these two variables. 

Considerable disparity exists with regards to the co-ingestion of protein to carbohydrate for promoting muscle glycogen repletion and little information is available in regards to the utilisation of glycogen during a repeated exhaustive exercise bout. We recently reported that muscle glycogen resynthesis was equally effective in restoring muscle glycogen when ingesting carbohydrate-protein when compared to the energy-matched carbohydrate only (in amounts suggested to elicit near-maximal reported muscle glycogen resynthesis rates), despite a greater insulin response in the former [22]. In accordance, a number of interesting questions remain with regards to post-exercise carbohydrate-protein ingestion. This relates to whether the augmented insulin response influences other insulin-sensitive tissues, such as the liver in modulating hepatic glucose output kinetics [170]. Another possible avenue of research in this field is to examine whether carbohydrate-protein increases the efficiency of glycogen storage from running relative to an isocaloric carbohydrate when provided in amounts less than that (<1.2 g·kg BM^−1^·h^−1^) suggested to maximise muscle glycogen resynthesis rates. This may be of particular relevance to many recreational exercisers as the aforementioned high carbohydrate intake guidelines are unlikely to be met by this population. 

## Figures and Tables

**Table 1:** Effects of carbohydrate ingestion during short-term recovery on repeated exercise capacity.

Study	Carbohydrate Intake (g·kg BM^−1^·h^−1^)	Recovery Time (h)	Timing of Ingestion (min)	Mode of Exercise	Repeated Exercise	Repeated Exercise Bout (min)
Fallowfield et al. [146]	0, 0.5	4	Immediately post-exercise, 120	Running	Time to exhaustion at 70% VO_2max_	40, 62 *
Fallowfield & Williams [152]	0.5, 1.5	4	Immediately post-exercise, 120	Running	Time to exhaustion at 70% VO_2max_	59, 58
Wong & Williams [154]	0.15, 0.53	4	30, 60, 90, 120, 150	Running	Time to exhaustion at 70% VO_2max_	65, 57
Wong et al. [151]	0, 0.9	4	30, 60, 90, 120, 150, 180	Running	Time to exhaustion at 70% VO_2max_	45, 69 *
Bilzon et al. [147]	0, 0.43	4	Immediately post-exercise, 60, 120, 180	Running	Time to exhaustion at 60% VO_2max_	45, 61 *
Casey et al. [25]	0, 0.25 G, 0.25 S	4	Immediately post-exercise	Cycling	Time to exhaustion at 70% VO_2max_	35, 40 G, 46 S
Betts et al. [23]	0.8, 1.1	4	Immediately post-exercise, 30, 60, 90, 120, 150, 180, 210	Running	Time to exhaustion at 70% VO_2max_	84, 100 *
Alghannam et al. [21]	0.3, 1.2	4	Immediately post-exercise, 30, 60, 90, 120, 150, 180, 210	Running	Time to exhaustion at 70% VO_2max_	48, 80 *

G, glucose; S, sucrose; * significantly greater than other treatment(s) (*p* ≤ 0.05).

**Table 2:** Effects of carbohydrate-protein ingestion during short-term recovery on repeated exercise capacity.

Study	Carbohydrate Intake (g·kg BM^−^^1^·h^−^^1^)	Protein Intake (g·kg BM^−^^1^·h^−^^1^)	Recovery Time (h)	Timing of Ingestion (min)	Mode of Exercise	Repeated Exercise	Repeated Exercise Bout (min)
Williams et al. [126]	0.15, 0.4	0, 0.1	4	Immediately post-exercise, 120	Running	Time to exhaustion at 85% VO_2max_	20, 31 ^*^
Millard-Stafford et al. [156]	0.6, 1.0, 0.8	0, 0, 0.2	2	Immediately post-exercise, 60	Running	Time to exhaustion at 90% VO_2max_	6, 6, 5
Betts et al. [157]	0.8, 0.8, 1.2, 1.2	0, 0.1, 0, 0.2	4	Immediately post-exercise, 30, 60, 90, 120, 150, 180, 210	Running	Time to exhaustion at 85% VO_2max_	18, 20, 15, 18
Karp et al. [158]	0.2, 0.5, 0.5 ^†^	0, 0.13, 0.13 ^†^	4	Immediately post-exercise, 120	Cycling	Time to exhaustion at 70% VO_2max_	41 ^*^, 29, 40 ^†,*^
Betts et al. [23]	0.8, 1.1, 0.8	0, 0, 0.3	4	Immediately post-exercise, 30, 60, 90, 120, 150, 180, 210	Running	Time to exhaustion at 70% VO_2max_	84, 91 ^*^, 100 ^*^
Thomas et al. [41]	0.2, 0.5, 0.4 ^†^	0, 0.13, 0.10 ^†^	4	Immediately post-exercise, 120	Cycling	Time to exhaustion at 70% VO_2max_	23, 21, 32 ^†,*^
Lunn et al. [40]	0.25, 0.20 ^†^	0, 0.05 ^†^	3	Immediately post-exercise	Running	Time to exhaustion at incline achieved at VO_2peak_ test	3, 4 ^†,*^
Richardson et al. [159]	1.5, 1.2	0, 0.3	3	Immediately post-exercise, 30, 60, 90, 120	Cycling	Time to exhaustion at 75% VO_2max_	25, 24
Alghannam et al. [22]	1.2,0.8	0, 0.4	4	Immediately post-exercise, 30, 60, 90, 120, 150, 180, 210	Running	Time to exhaustion at 70% VO_2max_	51, 49

^†^ Provided in the form of chocolate milk; * greater than other treatment(s) (*p* ≤ 0.05).

## References

[1] Reilly T., Ekblom B. (2005). The use of recovery methods post-exercise. J. Sports Sci..

[2] Ament W., Verkerke G.J. (2009). Exercise and fatigue. Sports Med..

[3] Enoka R.M., Duchateau J. (2008). Muscle fatigue: What, why and how it influences muscle function. J. Physiol..

[4] Marino F.E., Gard M., Drinkwater E.J. (2011). The limits to exercise performance and the future of fatigue research. Br. J. Sports Med..

[5] Phillips S. (2015). Fatigue in Sport and Exercise.

[6] Alghannam A.F., Jedrzejewski D., Tweddle M., Gribble H., Bilzon J.L., Betts J.A. (2016). Reliability of time to exhaustion treadmill running as a measure of human endurance capacity. Int. J. Sports Med..

[7] Thomas D.T., Erdman K.A., Burke L.M. (2016). American college of sports medicine joint position statement. nutrition and athletic performance. Med. Sci. Sports Exerc..

[8] Bergstrom J., Hermansen L., Hultman E., Saltin B. (1967). Diet, muscle glycogen and physical performance. Acta Physiol..

[9] Coyle E.F., Coggan A.R., Hemmert M.K., Ivy J.L. (1986). Muscle glycogen utilization during prolonged strenuous exercise when fed carbohydrate. J. Appl. Physiol..

[10] Hermansen L., Hultman E., Saltin B. (1967). Muscle glycogen during prolonged severe exercise. Acta Physiol. Scand..

[11] Coyle E.F., Jeukendrup A.E., Wagenmakers A.J., Saris W.H. (1997). Fatty acid oxidation is directly regulated by carbohydrate metabolism during exercise. Am. J. Physiol..

[12] Karelis A.D., Smith J.W., Passe D.H., Peronnet F. (2010). Carbohydrate administration and exercise performance: What are the potential mechanisms involved?. Sports Med..

[13] Tsintzas K., Williams C. (1998). Human muscle glycogen metabolism during exercise. Effect of carbohydrate supplementation. Sports Med..

[14] Jentjens R.L., Achten J., Jeukendrup A.E. (2004). High oxidation rates from combined carbohydrates ingested during exercise. Med. Sci. Sports Exerc..

[15] Jeukendrup A.E., Jentjens R. (2000). Oxidation of carbohydrate feedings during prolonged exercise: Current thoughts, guidelines and directions for future research. Sports Med..

[16] Tsintzas O.K., Williams C., Boobis L., Greenhaff P. (1996). Carbohydrate ingestion and single muscle fiber glycogen metabolism during prolonged running in men. J. Appl. Physiol..

[17] Betts J.A., Williams C. (2010). Short-term recovery from prolonged exercise exploring the potential for protein ingestion to accentuate the benefits of carbohydrate supplements. Sports Med..

[18] Tsintzas K., Williams C., Boobis L., Symington S., Moorehouse J., Garcia-Roves P., Nicholas C. (2003). Effect of carbohydrate feeding during recovery from prolonged running on muscle glycogen metabolism during subsequent exercise. Int. J. Sports Med..

[19] Berardi J.M., Price T.B., Noreen E.E., Lemon P.W.R. (2006). Postexercise muscle glycogen recovery enhanced with a carbohydrate-protein supplement. Med. Sci. Sports Exerc..

[20] Betts J.A., Williams C., Boobis L., Tsintzas K. (2008). Increased carbohydrate oxidation after ingesting carbohydrate with added protein. Med. Sci. Sports Exerc..

[21] Alghannam A.F., Jedrzejewski D., Tweddle M.G., Gribble H., Bilzon J., Thompson D., Tsintzas K., Betts J.A. (2016). Impact of muscle glycogen availability on the capacity for repeated exercise in man. Med. Sci. Sports Exerc..

[22] Alghannam A.F., Jedrzejewski D., Bilzon J., Thompson D., Tsintzas K., Betts J.A. (2016). Influence of post-exercise carbohydrate-protein ingestion on muscle glycogen metabolism in recovery and subsequent running exercise. Int. J. Sport Nutr. Exerc. Metab..

[23] Betts J., Williams C., Duffy K., Gunner F. (2007). The influence of carbohydrate and protein ingestion during recovery from prolonged exercise on subsequent endurance performance. J. Sports Sci..

[24] Ferguson-Stegall L., McCleave E.L., Ding Z., Doerner P.G., Wang B., Liao Y.H., Kammer L., Liu Y., Hwang J., Dessard B.M. (2011). Postexercise carbohydrate-protein supplementation improves subsequent exercise performance and intracellular signaling for protein synthesis. J. Strength Cond. Res..

[25] Casey A., Mann R., Banister K., Fox J., Morris P.G., Macdonald I.A., Greenhaff P.L. (2000). Effect of carbohydrate ingestion on glycogen resynthesis in human liver and skeletal muscle, measured by ^13^C MRS. Am. J. Physiol. Endocrinol. Metab..

[26] Allen D.G., Lamb G.D., Westerblad H. (2008). Skeletal muscle fatigue: Cellular mechanisms. Physiol. Rev..

[27] Nielsen J., Ortenblad N. (2013). Physiological aspects of the subcellular localization of glycogen in skeletal muscle. Appl. Physiol. Nutr. Metab..

[28] Nybo L., Nielsen B. (2001). Hyperthermia and central fatigue during prolonged exercise in humans. J. Appl. Physiol..

[29] Coyle E.F. (2000). Physical activity as a metabolic stressor. Am. J. Clin. Nutr..

[30] Sawka M.N., Noakes T.D. (2007). Does dehydration impair exercise performance?. Med. Sci. Sports Exerc..

[31] Rabinowitz D., Merimee T.J., Maffezzoli R., Burgess J.A. (1966). Patterns of hormonal release after glucose, protein, and glucose plus protein. Lancet.

[32] Van Loon L.J., Kruijshoop M., Verhagen H., Saris W.H., Wagenmakers A.J. (2000). Ingestion of protein hydrolysate and amino acid-carbohydrate mixtures increases postexercise plasma insulin responses in men. J. Nutr..

[33] Jentjens R.L., van Loon L.J., Mann C.H., Wagenmakers A.J., Jeukendrup A.E. (2001). Addition of protein and amino acids to carbohydrates does not enhance postexercise muscle glycogen synthesis. J. Appl. Physiol..

[34] Van Hall G., Shirreffs S.M., Calbet J.A. (2000). Muscle glycogen resynthesis during recovery from cycle exercise: No effect of additional protein ingestion. J. Appl. Physiol..

[35] Van Loon L.J., Saris W.H., Kruijshoop M., Wagenmakers A.J. (2000). Maximizing postexercise muscle glycogen synthesis: Carbohydrate supplementation and the application of amino acid or protein hydrolysate mixtures. Am. J. Clin. Nutr..

[36] Howarth K.R., Moreau N.A., Phillips S.M., Gibala M.J. (2009). Coingestion of protein with carbohydrate during recovery from endurance exercise stimulates skeletal muscle protein synthesis in humans. J. Appl. Physiol..

[37] Beelen M., Kranenburg J., Senden J.M., Kuipers H., van Loon L.J. (2012). Impact of caffeine and protein on postexercise muscle glycogen synthesis. Med. Sci. Sports Exerc..

[38] Carrithers J.A., Williamson D.L., Gallagher P.M., Godard M.P., Schulze K.E., Trappe S.W. (2000). Effects of postexercise carbohydrate-protein feedings on muscle glycogen restoration. J. Appl. Physiol..

[39] Ivy J.L., Goforth H.W., Damon B.M., McCauley T.R., Parsons E.C., Price T.B. (2002). Early postexercise muscle glycogen recovery is enhanced with a carbohydrate-protein supplement. J. Appl. Physiol..

[40] Lunn W.R., Pasiakos S.M., Colletto M.R., Karfonta K.E., Carbone J.W., Anderson J.M., Rodriguez N.R. (2012). Chocolate milk and endurance exercise recovery: Protein balance, glycogen, and performance. Med. Sci. Sports Exerc..

[41] Thomas K., Morris P., Stevenson E. (2009). Improved endurance capacity following chocolate milk consumption compared with 2 commercially available sport drinks. Appl. Physiol. Nutr. Metab..

[42] Jentjens R., Jeukendrup A. (2003). Determinants of post-exercise glycogen synthesis during short-term recovery. Sports Med..

[43] Horton T.J., Pagliassotti M.J., Hobbs K., Hill J.O. (1998). Fuel metabolism in men and women during and after long-duration exercise. J. Appl. Physiol..

[44] Kiens B., Richter E.A. (1998). Utilization of skeletal muscle triacylglycerol during postexercise recovery in humans. Am. J. Physiol..

[45] Egan B., Zierath J.R. (2013). Exercise metabolism and the molecular regulation of skeletal muscle adaptation. Cell Metab..

[46] Goforth H.W., Laurent D., Prusaczyk W.K., Schneider K.E., Petersen K.F., Shulman G.I. (2003). Effects of depletion exercise and light training on muscle glycogen supercompensation in men. Am. J. Physiol. Endocrinol. Metab..

[47] Melendez R., Melendez-Hevia E., Canela E.I. (1999). The fractal structure of glycogen: A clever solution to optimize cell metabolism. Biophys. J..

[48] Alonso M.D., Lomako J., Lomako W.M., Whelan W.J. (1995). A new look at the biogenesis of glycogen. FASEB J..

[49] Graham T.E., Yuan Z., Hill A.K., Wilson R.J. (2010). The regulation of muscle glycogen: The granule and its proteins. Acta Physiol..

[50] Jansson E. (1981). Acid soluble and insoluble glycogen in human skeletal muscle. Acta Physiol..

[51] James A.P., Barnes P.D., Palmer T.N., Fournier P.A. (2008). Proglycogen and macroglycogen: Artifacts of glycogen extraction?. Metabolism.

[52] Nielsen J., Holmberg H.C., Schroder H.D., Saltin B., Ortenblad N. (2011). Human skeletal muscle glycogen utilization in exhaustive exercise: Role of subcellular localization and fibre type. J. Physiol..

[53] Ortenblad N., Westerblad H., Nielsen J. (2013). Muscle glycogen stores and fatigue. J. Physiol..

[54] Marchand I., Tarnopolsky M., Adamo K.B., Bourgeois J.M., Chorneyko K., Graham T.E. (2007). Quantitative assessment of human muscle glycogen granules size and number in subcellular locations during recovery from prolonged exercise. J. Physiol..

[55] Nielsen J., Schroder H.D., Rix C.G., Ortenblad N. (2009). Distinct effects of subcellular glycogen localization on tetanic relaxation time and endurance in mechanically skinned rat skeletal muscle fibres. J. Physiol..

[56] Nielsen J., Cheng A.J., Ortenblad N., Westerblad H. (2014). Subcellular distribution of glycogen and decreased tetanic Ca^2+^ in fatigued single intact mouse muscle fibres. J. Physiol..

[57] Gejl K.D., Hvid L.G., Frandsen U., Jensen K., Sahlin K., Ortenblad N. (2014). Muscle glycogen content modifies SR Ca^2+^ release rate in elite endurance athletes. Med. Sci. Sports Exerc..

[58] Ortenblad N., Nielsen J., Saltin B., Holmberg H.C. (2011). Role of glycogen availability in sarcoplasmic reticulum Ca^2+^ kinetics in human skeletal muscle. J. Physiol..

[59] Chin E.R., Balnave C.D., Allen D.G. (1997). Role of intracellular calcium and metabolites in low-frequency fatigue of mouse skeletal muscle. Am. J. Physiol..

[60] Helander I., Westerblad H., Katz A. (2002). Effects of glucose on contractile function, [Ca^2+^]_i_, and glycogen in isolated mouse skeletal muscle. Am. J. Physiol. Cell Physiol..

[61] Maehlum S., Hostmark A.T., Hermansen L. (1977). Synthesis of muscle glycogen during recovery after prolonged severe exercise in diabetic and non-diabetic subjects. Scand. J. Clin. Lab. Investig..

[62] Price T.B., Rothman D.L., Taylor R., Avison M.J., Shulman G.I., Shulman R.G. (1994). Human muscle glycogen resynthesis after exercise: Insulin- dependent and -independent phases. J. Appl. Physiol..

[63] Price T.B., Perseghin G., Duleba A., Chen W., Chase J., Rothman D.L., Shulman R.G., Shulman G.I. (1996). NMR studies of muscle glycogen synthesis in insulin-resistant offspring of parents with non-insulin-dependent diabetes mellitus immediately after glycogen-depleting exercise. Proc. Natl. Acad. Sci. USA.

[64] Ivy J.L., Kuo C.H. (1998). Regulation of GLUT4 protein and glycogen synthase during muscle glycogen synthesis after exercise. Acta Physiol. Scand..

[65] Ivy J.L., Lee M.C., Brozinick J.T., Reed M.J. (1988). Muscle glycogen storage after different amounts of carbohydrate ingestion. J. Appl. Physiol..

[66] Goodyear L.J., Hirshman M.F., King P.A., Horton E.D., Thompson C.M., Horton E.S. (1990). Skeletal muscle plasma membrane glucose transport and glucose transporters after exercise. J. Appl. Physiol..

[67] Danforth W.H. (1965). Glycogen synthetase activity in skeletal muscle. Interconversion of two forms and control of glycogen synthesis. J. Biol. Chem..

[68] Cartee G.D., Young D.A., Sleeper M.D., Zierath J., Wallberg-Henriksson H., Holloszy J.O. (1989). Prolonged increase in insulin-stimulated glucose transport in muscle after exercise. Am. J. Physiol..

[69] Keizer H.A., Kuipers H., van Kranenburg G., Geurten P. (1987). Influence of liquid and solid meals on muscle glycogen resynthesis, plasma fuel hormone response, and maximal physical working capacity. Int. J. Sports Med..

[70] Lai Y.C., Zarrinpashneh E., Jensen J. (2010). Additive effect of contraction and insulin on glucose uptake and glycogen synthase in muscle with different glycogen contents. J. Appl. Physiol..

[71] Fisher J.S., Gao J., Han D.H., Holloszy J.O., Nolte L.A. (2002). Activation of amp kinase enhances sensitivity of muscle glucose transport to insulin. Am. J. Physiol. Endocrinol. Metab..

[72] Wojtaszewski J.F., MacDonald C., Nielsen J.N., Hellsten Y., Hardie D.G., Kemp B.E., Kiens B., Richter E.A. (2003). Regulation of 5′ AMP-activated protein kinase activity and substrate utilization in exercising human skeletal muscle. Am. J. Physiol. Endocrinol. Metab..

[73] Burke L.M., Kiens B., Ivy J.L. (2004). Carbohydrates and fat for training and recovery. J. Sports Sci..

[74] Beelen M., Burke L.M., Gibala M.J., van Loon L.J.C. (2010). Nutritional strategies to promote postexercise recovery. Int. J. Sport Nutr. Exerc. Metab..

[75] Tarnopolsky M.A., Bosman M., MacDonald J.R., Vandeputte D., Martin J., Roy B.D. (1997). Postexercise protein-carbohydrate and carbohydrate supplements increase muscle glycogen in men and women. J. Appl. Physiol..

[76] Roy B.D., Tarnopolsky M.A. (1998). Influence of differing macronutrient intakes on muscle glycogen resynthesis after resistance exercise. J. Appl. Physiol..

[77] Burke L.M., Hawley J.A., Wong S.H., Jeukendrup A.E. (2011). Carbohydrates for training and competition. J. Sports Sci..

[78] Jensen L., Gejl K.D., Ortenblad N., Nielsen J.L., Bech R.D., Nygaard T., Sahlin K., Frandsen U. (2015). Carbohydrate restricted recovery from long term endurance exercise does not affect gene responses involved in mitochondrial biogenesis in highly trained athletes. Physiol. Rep..

[79] Philp A., Hargreaves M., Baar K. (2012). More than a store: Regulatory roles for glycogen in skeletal muscle adaptation to exercise. Am. J. Physiol. Endocrinol. Metab..

[80] Bartlett J.D., Hawley J.A., Morton J.P. (2015). Carbohydrate availability and exercise training adaptation: Too much of a good thing?. Eur. J. Sport Sci..

[81] Maehlum S., Hermansen L. (1978). Muscle glycogen concentration during recovery after prolonged severe exercise in fasting subjects. Scand. J. Clin. Lab. Investig..

[82] Blom P.C., Hostmark A.T., Vaage O., Kardel K.R., Maehlum S. (1987). Effect of different post-exercise sugar diets on the rate of muscle glycogen synthesis. Med. Sci. Sports Exerc..

[83] Blom C.S. (1989). Post-exercise glucose uptake and glycogen synthesis in human muscle during oral or IV glucose intake. Eur. J. Appl. Physiol. Occup. Physiol..

[84] Zachwieja J.J., Costill D.L., Pascoe D.D., Robergs R.A., Fink W.J. (1991). Influence of muscle glycogen depletion on the rate of resynthesis. Med. Sci. Sports Exerc..

[85] Casey A., Short A.H., Hultman E., Greenhaff P.L. (1995). Glycogen resynthesis in human muscle fibre types following exercise-induced glycogen depletion. J. Physiol..

[86] Shearer J., Wilson R.J., Battram D.S., Richter E.A., Robinson D.L., Bakovic M., Graham T.E. (2005). Increases in glycogenin and glycogenin mrna accompany glycogen resynthesis in human skeletal muscle. Am. J. Physiol. Endocrinol. Metab..

[87] Jensen J., Aslesen R., Ivy J.L., Brors O. (1997). Role of glycogen concentration and epinephrine on glucose uptake in rat epitrochlearis muscle. Am. J. Physiol..

[88] Derave W., Lund S., Holman G.D., Wojtaszewski J., Pedersen O., Richter E.A. (1999). Contraction-stimulated muscle glucose transport and GLUT-4 surface content are dependent on glycogen content. Am. J. Physiol..

[89] Steensberg A., van Hall G., Keller C., Osada T., Schjerling P., Pedersen B.K., Saltin B., Febbraio M.A. (2002). Muscle glycogen content and glucose uptake during exercise in humans: Influence of prior exercise and dietary manipulation. J. Physiol..

[90] Kiens B., Raben A.B., Valeur A.-K., Richter E.A. (1990). Benefit of dietary simple carbohydrates on the early postexercise muscle glycogen repletion in male athletes. Med. Sci. Sports Exerc..

[91] Burke L.M., Collier G.R., Hargreaves M. (1993). Muscle glycogen storage after prolonged exercise: Effect of the glycemic index of carbohydrate feedings. J. Appl. Physiol..

[92] Wee S.L., Williams C., Tsintzas K., Boobis L. (2005). Ingestion of a high-glycemic index meal increases muscle glycogen storage at rest but augments its utilization during subsequent exercise. J. Appl. Physiol..

[93] Delarue J., Normand S., Pachiaudi C., Beylot M., Lamisse F., Riou J.P. (1993). The contribution of naturally labelled ^13^C fructose to glucose appearance in humans. Diabetologia.

[94] Nilsson L.H., Hultman E. (1974). Liver and muscle glycogen in man after glucose and fructose infusion. Scand. J. Clin. Lab. Investig..

[95] Bergstrom J., Hultman E. (1967). Synthesis of muscle glycogen in man after glucose and fructose infusion. Acta Med. Scand..

[96] Van den Bergh A.J., Houtman S., Heerschap A., Rehrer N.J., van den Boogert H.J., Oeseburg B., Hopman M.T. (1996). Muscle glycogen recovery after exercise during glucose and fructose intake monitored by ^13^C-NMR. J. Appl. Physiol..

[97] Wallis G.A., Hulston C.J., Mann C.H., Roper H.P., Tipton K.D., Jeukendrup A.E. (2008). Postexercise muscle glycogen synthesis with combined glucose and fructose ingestion. Med. Sci. Sports Exerc..

[98] Trommelen J., Beelen M., Pinckaers P.J., Senden J.M., Cermak N.M., van Loon L.J. (2016). Fructose coingestion does not accelerate postexercise muscle glycogen repletion. Med. Sci. Sports Exerc..

[99] Fuchs C.J., Gonzalez J.T., Beelen M., Cermak N.M., Smith F.E., Thelwall P.E., Taylor R., Trenell M.I., Stevenson E.J., van Loon L.J. (2016). Sucrose ingestion after exhaustive exercise accelerates liver, but not muscle glycogen repletion compared with glucose ingestion in trained athletes. J. Appl. Physiol..

[100] Gonzalez J.T., Fuchs C.J., Betts J.A., van Loon L.J. (2017). Glucose plus fructose ingestion for post-exercise recovery-greater than the sum of its parts?. Nutrients.

[101] Maunder E., Podlogar T., Wallis G.A. (2017). Postexercise fructose-maltodextrin ingestion enhances subsequent endurance capacity. Med. Sci. Sports Exerc..

[102] Reed M.J., Brozinick J.T., Lee M.C., Ivy J.L. (1989). Muscle glycogen storage postexercise: Effect of mode of carbohydrate administration. J. Appl. Physiol..

[103] Wallis G.A., Wittekind A. (2013). Is there a specific role for sucrose in sports and exercise performance?. Int. J. Sport Nutr. Exerc. Metab..

[104] Lecoultre V., Benoit R., Carrel G., Schutz Y., Millet G.P., Tappy L., Schneiter P. (2010). Fructose and glucose co-ingestion during prolonged exercise increases lactate and glucose fluxes and oxidation compared with an equimolar intake of glucose. Am. J. Clin. Nutr..

[105] Ivy J.L. (2013). The regulation and synthesis of muscle glycogen by means of nutrient intervention. Encycl. Sports Med..

[106] Levenhagen D.K., Gresham J.D., Carlson M.G., Maron D.J., Borel M.J., Flakoll P.J. (2001). Postexercise nutrient intake timing in humans is critical to recovery of leg glucose and protein homeostasis. Am. J. Physiol. Endocrinol. Metab..

[107] Ivy J.L. (2001). Dietary strategies to promote glycogen synthesis after exercise. Can. J. Appl. Physiol..

[108] Parkin J.A., Carey M.F., Martin I.K., Stojanovska L., Febbraio M.A. (1997). Muscle glycogen storage following prolonged exercise: Effect of timing of ingestion of high glycemic index food. Med. Sci. Sports Exerc..

[109] Jensen J., Jebens E., Brennesvik E.O., Ruzzin J., Soos M.A., Engebretsen E.M., O’Rahilly S., Whitehead J.P. (2006). Muscle glycogen inharmoniously regulates glycogen synthase activity, glucose uptake, and proximal insulin signaling. Am. J. Physiol. Endocrinol. Metab..

[110] Costill D.L., Sherman W.M., Fink W.J., Maresh C., Witten M., Miller J.M. (1981). The role of dietary carbohydrates in muscle glycogen resynthesis after strenuous running. Am. J. Clin. Nutr..

[111] Burke L.M., Collier G.R., Davis P.G., Fricker P.A., Sanigorski A.J., Hargreaves M. (1996). Muscle glycogen storage after prolonged exercise: Effect of the frequency of carbohydrate feedings. Am. J. Clin. Nutr..

[112] Ivy J.L. (1998). Glycogen resynthesis after exercise: Effect of carbohydrate intake. Int. J. Sports Med..

[113] Doyle J.A., Sherman W.M., Strauss R.L. (1993). Effects of eccentric and concentric exercise on muscle glycogen replenishment. J. Appl. Physiol..

[114] Floyd J.C., Fajans S.S., Conn J.W., Knopf R.F., Rull J. (1966). Insulin secretion in response to protein ingestion. J. Clin. Investig..

[115] Floyd J.C., Fajans S.S., Pek S., Thiffault C.A., Knopf R.F., Conn J.W. (1970). Synergistic effect of certain amino acid pairs upon insulin secretion in man. Diabetes.

[116] Gonzalez J.T., Fuchs C.J., Smith F.E., Thelwall P.E., Taylor R., Stevenson E.J., Trenell M.I., Cermak N.M., van Loon L.J. (2015). Ingestion of glucose or sucrose prevents liver but not muscle glycogen depletion during prolonged endurance-type exercise in trained cyclists. Am. J. Physiol. Endocrinol. Metab..

[117] Mace O.J., Schindler M., Patel S. (2012). The regulation of K- and L -cell activity by GLUT2 and the calcium-sensing receptor casr in rat small intestine. J. Physiol..

[118] Koopman R., Crombach N., Gijsen A.P., Walrand S., Fauquant J., Kies A.K., Lemosquet S., Saris W.H., Boirie Y., van Loon L.J. (2009). Ingestion of a protein hydrolysate is accompanied by an accelerated in vivo digestion and absorption rate when compared with its intact protein. Am. J. Clin. Nutr..

[119] Morifuji M., Ishizaka M., Baba S., Fukuda K., Matsumoto H., Koga J., Kanegae M., Higuchi M. (2010). Comparison of different sources and degrees of hydrolysis of dietary protein: Effect on plasma amino acids, dipeptides, and insulin responses in human subjects. J. Agric. Food Chem..

[120] Reitelseder S., Agergaard J., Doessing S., Helmark I.C., Lund P., Kristensen N.B., Frystyk J., Flyvbjerg A., Schjerling P., van Hall G. (2011). Whey and casein labeled with L-[1-^13^C] leucine and muscle protein synthesis: Effect of resistance exercise and protein ingestion. Am. J. physiol. Metab..

[121] Van Loon L.J., Saris W.H., Verhagen H., Wagenmakers A.J. (2000). Plasma insulin responses after ingestion of different amino acid or protein mixtures with carbohydrate. Am. J. Clin. Nutr..

[122] Di Camillo B., Eduati F., Nair S.K., Avogaro A., Toffolo G.M. (2014). Leucine modulates dynamic phosphorylation events in insulin signaling pathway and enhances insulin-dependent glycogen synthesis in human skeletal muscle cells. BMC Cell Biol..

[123] Morifuji M., Aoyama T., Nakata A., Sambongi C., Koga J., Kurihara K., Kanegae M., Suzuki K., Higuchi M. (2012). Post-exercise ingestion of different amounts of protein affects plasma insulin concentration in humans. Eur. J. Sport Sci..

[124] Zawadzki K., Yaspelkis B.B., Ivy J. (1992). Carbohydrate-protein complex increases the rate of muscle glycogen storage after exercise. J. Appl. Physiol..

[125] Kaastra B., Manders R.J., van Breda E., Kies A., Jeukendrup A.E., Keizer H.A., Kuipers H., van Loon L.J. (2006). Effects of increasing insulin secretion on acute postexercise blood glucose disposal. Med. Sci. Sports Exerc..

[126] Williams M.B., Raven P.B., Fogt D.L., Ivy J.L. (2003). Effects of recovery beverages on glycogen restoration and endurance exercise performance. J. Strength Cond. Res..

[127] Morifuji M., Kanda A., Koga J., Kawanaka K., Higuchi M. (2010). Post-exercise carbohydrate plus whey protein hydrolysates supplementation increases skeletal muscle glycogen level in rats. Amino Acid..

[128] Hara D., Morrison P.J., Ding Z., Ivy J.L. (2011). Effect of carbohydrate-protein supplementation postexercise on rat muscle glycogen synthesis and phosphorylation of proteins controlling glucose storage. Metabolism.

[129] Rotman S., Slotboom J., Kreis R., Boesch C., Jequier E. (2000). Muscle glycogen recovery after exercise measured by ^13^C-magnetic resonance spectroscopy in humans: Effect of nutritional solutions. Magn. Reson. Mater. Phys. Biol. Med..

[130] Detko E., O’Hara J.P., Thelwall P.E., Smith F.E., Jakovljevic D.G., King R.F., Trenell M.I. (2013). Liver and muscle glycogen repletion using ^13^C magnetic resonance spectroscopy following ingestion of maltodextrin, galactose, protein and amino acids. Br. J. Nutr..

[131] Wang B., Ding Z., Wang W., Hwang J., Liao Y.H., Ivy J.L. (2015). The effect of an amino acid beverage on glucose response and glycogen replenishment after strenuous exercise. Eur. J. Appl. Physiol..

[132] Cogan K.E., Evans M., Iuliano E., Melvin A., Susta D., Neff K., de Vito G., Egan B. (2018). Co-ingestion of protein or a protein hydrolysate with carbohydrate enhances anabolic signaling, but not glycogen resynthesis, following recovery from prolonged aerobic exercise in trained cyclists. Eur. J. Appl. Physiol..

[133] Taylor R., Price T.B., Rothman D.L., Shulman R.G., Shulman G.I. (1992). Validation of ^13^C NMR measurement of human skeletal muscle glycogen by direct biochemical assay of needle biopsy samples. Magn. Reson. Med..

[134] Kristiansen S., Asp S., Richter E.A. (1996). Decreased muscle GLUT-4 and contraction-induced glucose transport after eccentric contractions. Am. J. Physiol..

[135] Asp S., Watkinson A., Oakes N.D., Kraegen E.W. (1997). Prior eccentric contractions impair maximal insulin action on muscle glucose uptake in the conscious rat. J. Appl. Physiol..

[136] Costill D.L., Pascoe D.D., Fink W.J., Robergs R.A., Barr S.I., Pearson D. (1990). Impaired muscle glycogen resynthesis after eccentric exercise. J. Appl. Physiol..

[137] Doi M., Yamaoka I., Nakayama M., Mochizuki S., Sugahara K., Yoshizawa F. (2005). Isoleucine, a blood glucose-lowering amino acid, increases glucose uptake in rat skeletal muscle in the absence of increases in amp-activated protein kinase activity. J. Nutr..

[138] Bernard J.R., Liao Y.H., Hara D., Ding Z., Chen C.Y., Nelson J.L., Ivy J.L. (2011). An amino acid mixture improves glucose tolerance and insulin signaling in sprague-dawley rats. Am. J. Physiol. Endocrinol. Metab..

[139] Kleinert M., Liao Y.H., Nelson J.L., Bernard J.R., Wang W., Ivy J.L. (2011). An amino acid mixture enhances insulin-stimulated glucose uptake in isolated rat epitrochlearis muscle. J. Appl. Physiol..

[140] Spiller G.A., Jensen C.D., Pattison T.S., Chuck C.S., Whittam J.H., Scala J. (1987). Effect of protein dose on serum glucose and insulin response to sugars. Am. J. Clin. Nutr..

[141] Boden G., Cheung P., Stein T.P., Kresge K., Mozzoli M. (2002). FFA cause hepatic insulin resistance by inhibiting insulin suppression of glycogenolysis. Am. J. Physiol. Endocrinol. Metab..

[142] Ahlborg B., Bergstrom J., Ekelund L., Hultman E. (1967). Muscle glycogen and muscle electrolytes during prolonged physical exercise. Acta Physiol. Scand..

[143] Nicholas C.W., Green P.A., Hawkins R.D., Williams C. (1997). Carbohydrate intake and recovery of intermittent running capacity. Int. J. Sport Nutr..

[144] Costill D.L., Flynn M.G., Kirwan J.P., Houmard J.A., Mitchell J.B., Thomas R., Park S.H. (1988). Effects of repeated days of intensified training on muscle glycogen and swimming performance. Med. Sci. Sports Exerc..

[145] Yeo W.K., Paton C.D., Garnham A.P., Burke L.M., Carey A.L., Hawley J.A. (2008). Skeletal muscle adaptation and performance responses to once a day versus twice every second day endurance training regimens. J. Appl. Physiol..

[146] Fallowfield J.L., Williams C., Singh R. (1995). The influence of ingesting a carbohydrate-electrolyte beverage during 4 hours of recovery on subsequent endurance capacity. Int. J. Sport Nutr..

[147] Bilzon J.L., Allsopp A.J., Williams C. (2000). Short-term recovery from prolonged constant pace running in a warm environment: The effectiveness of a carbohydrate-electrolyte solution. Eur. J. Appl. Physiol..

[148] Lee J.K.W., Nio A.Q.X., Ang W.H., Law L.Y.L., Lim C.L. (2011). Effects of ingesting a sports drink during exercise and recovery on subsequent endurance capacity. Eur. J. Sport Sci..

[149] Taylor C., Higham D., Close G.L., Morton J.P. (2011). The effect of adding caffeine to postexercise carbohydrate feeding on subsequent high-intensity interval-running capacity compared with carbohydrate alone. Int. J. Sport Nutr. Exerc. Metab..

[150] Nybo L. (2010). CNS fatigue provoked by prolonged exercise in the heat. Front. Biosci..

[151] Wong S.H., Williams C., Adams N. (2000). Effects of ingesting a large volume of carbohydrate-electrolyte solution on rehydration during recovery and subsequent exercise capacity. Int. J. Sport Nutr. Exerc. Metab..

[152] Fallowfield J.L., Williams C. (1997). The influence of a high carbohydrate intake during recovery from prolonged, constant-pace running. Int. J. Sport Nutr..

[153] Siu P.M., Wong S.H., Morris J.G., Lam C.W., Chung P.K., Chung S. (2004). Effect of frequency of carbohydrate feedings on recovery and subsequent endurance run. Med. Sci. Sports Exerc..

[154] Wong S.H., Williams C. (2000). Influence of different amounts of carbohydrate on endurance running capacity following short term recovery. Int. J. Sports Med..

[155] Hopkins W.G., Schabort E.J., Hawley J.A. (2001). Reliability of power in physical performance tests. Sports Med..

[156] Millard-Stafford M., Warren G.L., Thomas L.M., Doyle J.A., Snow T., Hitchcock K. (2005). Recovery from run training: Efficacy of a carbohydrate-protein beverage?. Int. J. Sport Nutr. Exerc. Metab..

[157] Betts J.A., Stevenson E., Williams C., Sheppard C., Grey E., Griffin J. (2005). Recovery of endurance running capacity: Effect of carbohydrate-protein mixtures. Int. J. Sport Nutr. Exerc. Metab..

[158] Karp J.R., Johnston J.D., Tecklenburg S., Mickleborough T.D., Fly A.D., Stager J.M. (2006). Chocolate milk as a post-exercise recovery aid. Int. J. Sport Nutr. Exerc. Metab..

[159] Richardson K.L., Coburn J.W., Beam W.C., Brown L.E. (2012). Effects of isocaloric carbohydrate vs. carbohydrate-protein supplements on cycling time to exhaustion. J. Strength Cond. Res..

[160] Pedersen D.J., Lessard S.J., Coffey V.G., Churchley E.G., Wootton A.M., Ng T., Watt M.J., Hawley J.A. (2008). High rates of muscle glycogen resynthesis after exhaustive exercise when carbohydrate is coingested with caffeine. J. Appl. Physiol..

[161] Berardi J.M., Noreen E.E., Lemon P.W. (2008). Recovery from a cycling time trial is enhanced with carbohydrate-protein supplementation vs. isoenergetic carbohydrate supplementation. J. Int. Soc. Sports Nutr..

[162] Wagenmakers A.J. (1998). Muscle amino acid metabolism at rest and during exercise: Role in human physiology and metabolism. Exerc. Sport Sci. Rev..

[163] Gibala M.J., Gonzalez-Alonso J., Saltin B. (2002). Dissociation between muscle tricarboxylic acid cycle pool size and aerobic energy provision during prolonged exercise in humans. J. Physiol..

[164] Meeusen R. (2014). Exercise, nutrition and the brain. Sports Med..

[165] Blomstrand E., Hassmen P., Ek S., Ekblom B., Newsholme E.A. (1997). Influence of ingesting a solution of branched-chain amino acids on perceived exertion during exercise. Acta Physiol. Scand..

[166] Alghannam A.F. (2011). Carbohydrate-protein ingestion improves subsequent running capacity towards the end of a football-specific intermittent exercise. Appl. Physiol. Nutr. Metab..

[167] Van Hall G., Raaymakers J.S., Saris W.H., Wagenmakers A.J. (1995). Ingestion of branched-chain amino acids and tryptophan during sustained exercise in man: Failure to affect performance. J. Physiol..

[168] Madsen K., MacLean D.A., Kiens B., Christensen D. (1996). Effects of glucose, glucose plus branched-chain amino acids, or placebo on bike performance over 100 km. J. Appl. Physiol..

[169] Morifuji M., Kanda A., Koga J., Kawanaka K., Higuchi M. (2011). Preexercise ingestion of carbohydrate plus whey protein hydrolysates attenuates skeletal muscle glycogen depletion during exercise in rats. Nutrition.

[170] Marmy-Conus N., Fabris S., Proietto J., Hargreaves M. (1996). Preexercise glucose ingestion and glucose kinetics during exercise. J. Appl. Physiol..

